# Prevalence and extent of infarct and microvascular obstruction following different reperfusion therapies in ST-elevation myocardial infarction

**DOI:** 10.1186/1532-429X-16-38

**Published:** 2014-05-27

**Authors:** Jamal N Khan, Naveed Razvi, Sheraz A Nazir, Anvesha Singh, Nicholas GD Masca, Anthony H Gershlick, Iain Squire, Gerry P McCann

**Affiliations:** 1 Department of Cardiovascular Sciences, University of Leicester, Glenfield Hospital, Groby Road, Leicester LE3 9QP, UK; 2 NIHR Leicester Cardiovascular Biomedical Research Unit, Glenfield Hospital, Groby Road, Leicester LE3 9QP, UK

**Keywords:** Cardiovascular magnetic resonance, Myocardial infarction, Microvascular obstruction, Primary angioplasty, Thrombolysis, Reperfusion, Ischaemia-reperfusion injury, Reperfusion injury

## Abstract

**Background:**

Microvascular obstruction (MVO) describes suboptimal tissue perfusion despite restoration of infarct-related artery flow. There are scarce data on Infarct Size (IS) and MVO in relation to the mode and timing of reperfusion. We sought to characterise the prevalence and extent of microvascular injury and IS using Cardiovascular magnetic resonance (CMR), in relation to the mode of reperfusion following acute ST-Elevation Myocardial Infarction (STEMI).

**Methods:**

CMR infarct characteristics were measured in 94 STEMI patients (age 61.0 ± 13.1 years) at 1.5 T. Seventy-three received reperfusion therapy: primary percutaneous coronary-intervention (PPCI, n = 47); thrombolysis (n = 12); rescue PCI (R-PCI, n = 8), late PCI (n = 6). Twenty-one patients presented late (>12 hours) and did not receive reperfusion therapy.

**Results:**

IS was smaller in PPCI (19.8 ± 13.2% of LV mass) and thrombolysis (15.2 ± 10.1%) groups compared to patients in the late PCI (40.0 ± 15.6%) and R-PCI (34.2 ± 18.9%) groups, p <0.001. The prevalence of MVO was similar across all groups and was seen at least as frequently in the non-reperfused group (15/21, [76%] v 33/59, [56%], p = 0.21) and to a similar magnitude (1.3 (0.0-2.8) v 0.4 [0.0-2.9]% LV mass, p = 0.36) compared to patients receiving early reperfusion therapy. In the 73 reperfused patients, time to reperfusion, ischaemia area at risk and TIMI grade post-PCI were the strongest independent predictors of IS and MVO.

**Conclusions:**

In patients with acute STEMI, CMR-measured MVO is not exclusive to reperfusion therapy and is primarily related to ischaemic time. This finding has important implications for clinical trials that use CMR to assess the efficacy of therapies to reduce reperfusion injury in STEMI.

## Background

In the setting of acute ST-segment elevation myocardial infarction (STEMI), microvascular obstruction (MVO) describes suboptimal tissue perfusion despite restoration of flow in the infarct-related artery (IRA). MVO is generally thought to be related primarily to reperfusion injury [1-3]. Cardiovascular magnetic resonance (CMR) provides unique characterisation of myocardial injury post STEMI [4].

CMR-measured MVO correlates strongly with ST-segment resolution in patients undergoing primary percutaneous coronary intervention (PPCI) but relatively weakly with myocardial blush-grade and poorly with TIMI flow [5]. Larger infarcts on CMR are consistently associated with larger ventricular volumes, lower ejection fraction and greater MVO [6], which occurs in 40-60% of patients treated by primary percutaneous coronary intervention (PPCI). CMR-derived infarct size (IS) [4,7] and MVO [8,9] are powerful predictors of adverse remodelling and prognosis post STEMI.

The European Society of Cardiology (ESC) [10] advocates four reperfusion strategies for acute STEMI: PPCI, thrombolysis, rescue coronary angioplasty (R-PCI) and late PCI (>12 hours after symptoms). There is a paucity of data on the prevalence and extent of MVO following STEMI, with different reperfusion therapies [11,12], and in particular in patients who do not receive any reperfusion therapy.

This study aimed to characterise the prevalence and extent of microvascular injury (MVO) and IS using CMR, in relation to the mode of reperfusion following STEMI.

## Methods

### Subjects and reperfusion therapy

**Figure 1:**
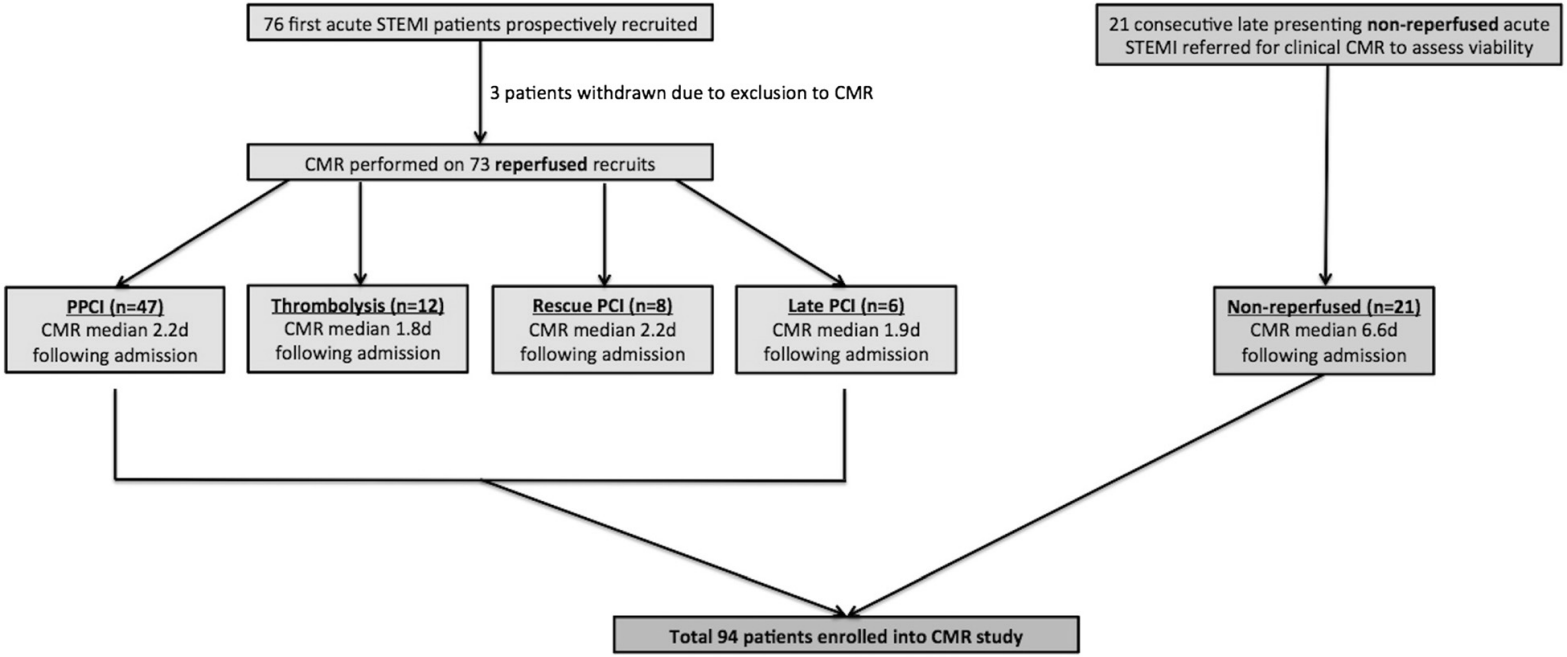
Study recruitment.

Ninety-seven patients presenting to a single regional cardiac centre with a first acute STEMI from Jan 2010 to April 2012 were included. Diagnosis of STEMI was made according to ACCF/AHA and ESC definitions [10]. Seventy-six patients who received one of the four advocated reperfusion strategies were recruited prospectively in a study assessing left ventricular (LV) remodeling (Figure 1). Three patients were excluded due to inability to complete CMR. The remaining 73 patients were treated as follows: PPCI (n = 47), thrombolysis (n = 12), R-PCI (n = 8), late PCI (n = 6). Reperfusion therapy was decided at the point of first medical contact according to local guidelines. Late PCI patients underwent PCI >12 hours after symptom onset (TTR) in the presence of electrocardiographic or clinical evidence of ongoing ischaemia. Twenty-one consecutive STEMI patients who presented late (>12 hours after symptom onset) and were symptom-free on arrival and did not receive reperfusion therapy formed the non-reperfused cohort. These patients underwent clinical CMR to assess myocardial viability. The local research ethics committee approved the study and prospectively recruited patients provided written consent prior to participation.

‘Early-reperfused’ patients were defined as those undergoing successful initial reperfusion within 12 hours of symptoms (PPCI, successful thrombolysis). Thrombolysis was performed in patients presenting to non-PCI capable regional hospitals using tissue plasminogen-activator analogues. Successful thrombolysis was defined as symptom resolution and ≥50% resolution of ST-segment elevation within 90 minutes, and was followed by transfer to our centre for coronary angiography. Immediate transfer for R-PCI was undertaken for thrombolysis failure. Time to reperfusion (TTR) was measured as the time between symptom onset and successful restoration of IRA flow for PCI-related revascularisation, and time until administration of successful thrombolytic therapy for thrombolysed patients.

### Angiographic assessment

The Thrombolysis in Myocardial Infarction (TIMI) scoring system was used to quantify angiographic IRA flow [13]. The degree of collateral flow to the IRA territory was quantified using the Rentrop Score (Grade 0: absent visible collateral flow; Grade 1: IRA side-branches only filled; Grade 2: partial filling of main IRA vessel; Grade 3: IRA completely filled by collaterals) [14].

### CMR image acquisition

**Figure 2:**
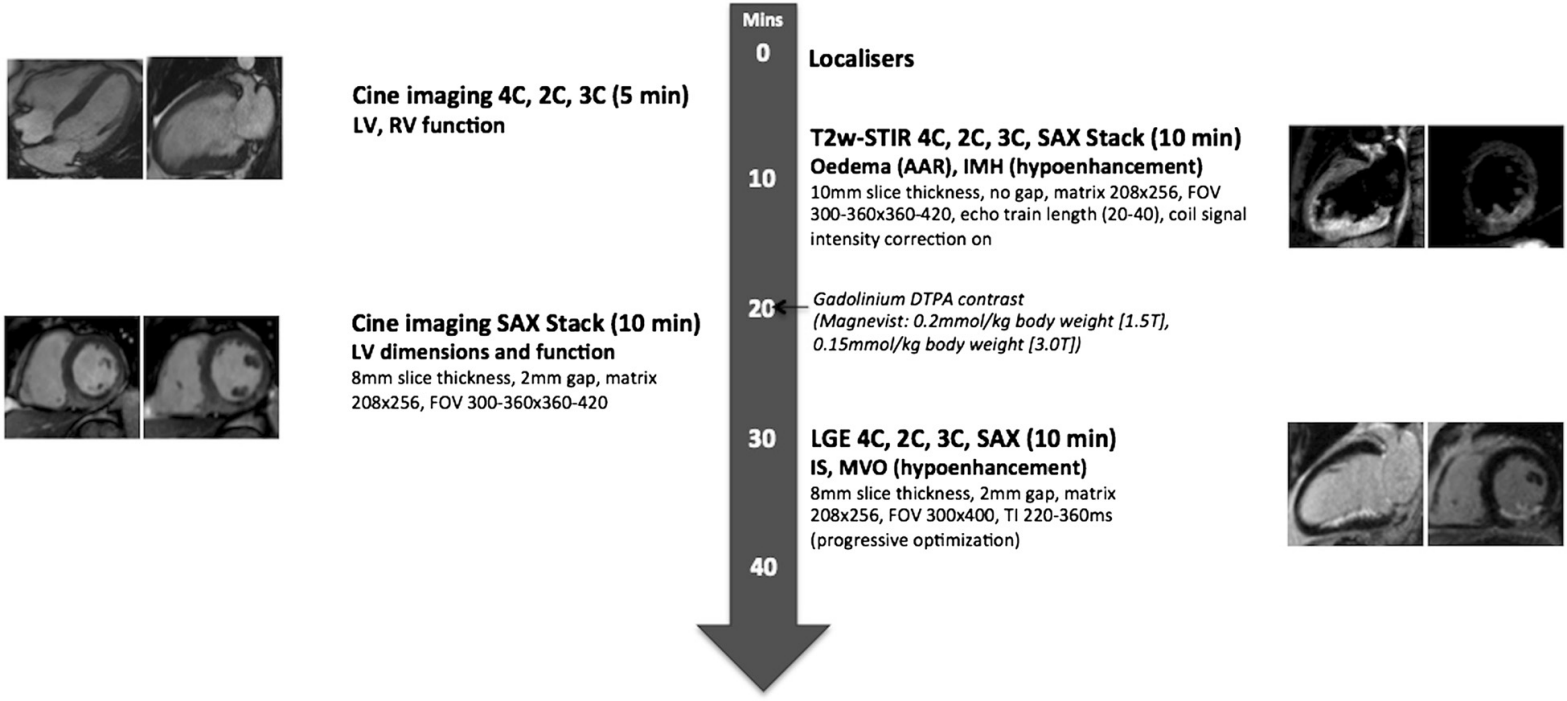
**CMR protocol.** SAX = short-axis, LV = left ventricle, RV = right ventricle, T2w-STIR = T2-weighted short-tau inversion recovery, AAR = area at risk, IMH = intramyocardial haemorrhage, LGE = late gadolinium enhancement, MVO = microvascular obstruction.

CMR was performed on all subjects during the index admission on a 1.5 T scanner (Siemens Avanto, Erlangen, Germany) with retrospective electrocardiogram gating and a 6-channel phased-array cardiac receiver coil supervised by a cardiologist with a subspecialist interest in CMR (Figure 2). Cine imaging with steady-state free precession and Late Gadolinium Enhancement (LGE) imaging were performed in long-axis views and contiguous short-axis slices covering the entire LV. LGE images were acquired 10–15 minutes after contrast administration using a segmented inversion-recovery gradient-echo sequence. The inversion time was progressively adjusted to null unaffected myocardium. T2-weighted short-tau inversion recovery (T2w-STIR) imaging with coil signal intensity correction was performed on the 73 prospectively recruited reperfused subjects and not on the 21 non-reperfused patients since they underwent a routine clinical CMR protocol to assess for viability.

### CMR image analysis

Analysis was performed offline blinded to patient details using QMass 7.1 (Medis, Leiden, Netherlands) by two experienced observers (JNK, NAR with 3 years CMR experience each). LV volumes and function were calculated as previously described [5]. Ischaemic area at risk ([AAR] oedema) was defined semi-automatically as areas of hyperenhancement ≥2 standard deviations above the signal intensity of unaffected myocardium. Infarct zone was defined semi-automatically on LGE imaging using the Full-Width Half-Maximum (FWHM) technique [15]. MVO was defined as areas of hypoenhancement within the infarct zone and was included in the assessed IS. AAR, IS and MVO were expressed as a percentage of LV end-diastolic mass (%LVM) and LV volumes were indexed by body-surface area.

### Statistical analysis

Normality was assessed using the Kolmogorov-Smirnov test, histograms and Q-Q plots. Normally distributed data were expressed as mean ± standard deviation and analysed using ANOVA and independent t-tests. Non-normally distributed variables were expressed as median (25%-75% interquartile range) and analysed using Mann–Whitney U-tests. Chi-squared analysis was used to compare MVO prevalence between cohorts. The association between time from symptom onset to revascularisation (TTR), AAR, time to CMR after admission, left anterior descending artery infarct related artery (LAD IRA), TIMI flow pre and post-PCI and revascularisation method with IS and MVO were assessed for reperfused patients using univariate regression. Predictors with p < 0.1 underwent stepwise multivariate analysis. Since categorical and continuous variables were used, the strength of variables was expressed according to their p-value. CMR markers were corrected for TTR using ANCOVA. Reproducibility of CMR analysis was assessed using two-way mixed-effect intraclass correlation coefficient for absolute agreement (ICC) for a subset of 10 randomly chosen studies. Statistical tests were performed on SPSS version 20. p < 0.05 was considered significant.

## Results

### Baseline characteristics

Baseline demographics and angiography findings are summarised in Table 1. Diabetes was more prevalent in the non-reperfused group and TTR was longer in late PCI patients than the other groups. TIMI flow grade in successfully thrombolysed patients at the start of angiography was higher than in the other cohorts. Fifteen (71.4%) non-reperfused patients underwent coronary angiography (pre-CMR in 6 patients; post-CMR in 9). In 12 (80%) of these patients, TIMI flow-grade was abnormal (TIMI-0 in 6 patients, TIMI-1 in 4, TIMI-2 in 2).

**Table 1:** Baseline demographics and angiographic data by reperfusion therapy

**Variable**	**Group 1, n = 47**	**Group 2, n = 12**	**Group 3, n = 8**	**Group 4, n = 6**	**Group 5, n = 21**	**p**
	**(PPCI)**	**(Thrombolysis)**	**(Rescue-PCI)**	**(Late PCI)**	**(Non-reperfused)**	
Age (years)	60.5 ± 12.3	59.3 ± 10.6	59.5 ± 12.5	54.7 ± 12.1	65.6 ± 16.2	0.37
Male sex (n,%)	42 (89.7)	11 (91.7)	8 (100)	5 (83.3)	16 (76.2)	0.21
Current smoking (n,%)	23 (48.9)	6 (50)	3 (37.5)	1 (16.7)	9 (42.9)	0.64
Diabetes (n,%)	2 (4.3)	1 (0)	0 (0)	0 (0)	6 (28.6)	*0.01*
Angina (n,%)	2 (4.3)	1 (8.3)	0 (0)	0 (0)	5 (23.8)	0.07
TTR (mins)	150 (120–240)	210 (75–300)	285 (211.25-345)	1113 (810–1342)	n/a	*<0.001*
Peak CK (iU/L)	875 (415.3-2061)	1034 (334.5-1384)	3002 (758–5045.5)	2633 (1073.3-5852)	1033 (87.8-2220.3)	0.88
**Angiography**					**(n = 15)**	
LAD IRA (n,%)	19 (40.4)	6 (50)	4 (50)	6 (100)	8 (53.3)	0.10
LCX IRA (n,%)	8 (17.0)	0 (0)	1 (12.5)	0 (0)	4 (26.7)	0.10
RCA IRA (n,%)	20 (42.6)	6 (50)	3 (37.5)	0 (0)	3 (20.0)	0.10
Multi-vessel disease (n,%)	16 (34)	4 (33.3)	1 (12.5)	0 (0)	5 (33.3)	0.38
Rentrop Score	0 (0–1)	0 (0–0)	0 (0–0)	0 (0-)	0 (0–0)	0.51
Rentrop B (Grd 2–3, n,%)	6 (13.3)	0 (0)	0 (0)	1 (16.7)	1 (6.7)	0.50
TIMI flow pre 0-II (n,%)	43 (95.6)	6 (83.3)	8 (100)	5 (83.3)	12 (80.0)	0.27
TIMI flow post III (n,%)	31 (68.9)	9 (58.3)	4 (50)	4 (66.7)	n/a	0.82
GPIIb/IIIa inhibitor use	18 (41.9)	0 (0)	3 (37.5)	2 (40)	n/a	0.39
Thrombectomy catheter?	20 (42.6)	0 (0)	5 (62.5)	1 (16.7)	n/a	*0.01*

Angiographic data available for 88/94 patients (angiography not performed in 6/21 non-reperfused patients).

TTR = time from symptom onset to revascularisation, PPCI = primary percutaneous coronary angioplasty, IRA = infarct-related artery, LAD = left anterior descending artery, LCX = left circumflex artery, RCA = right coronary artery, TIMI = thrombolysis in myocardial infarction, (pre) = TIMI score at start of coronary angiogram, (post) = TIMI score post-PCI, GPIIb/IIIa = glycoprotein IIb/IIIa inhibitor.

### CMR data

CMR data are shown in Table 2. The median time from admission to CMR was longer in the non-reperfused cohort compared with the other reperfusion strategies.

**Table 2:** CMR data by reperfusion therapy

**Variable**	**Group 1, n = 47 (PPCI)**	**Group 2, n = 12 (Thrombolysis)**	**Group 3, n = 8 (Rescue-PCI)**	**Group 4, n = 6 (Late PCI)**	**Group 5, n = 21 (Non-reperfused)**	**p**	**p (corrected for TTR)**
Time admission-CMR (d)	1.8 (1.1-2.6)	2.2 (1.3-2.6)	1.9 (1.4-3.8)	1.9 (1.5-3.6)	6.6 (4.8-11.0)	*<0.001*	--
LVEDVI (ml/m2)	91.6 (84.9-102.7)	83.8 (76.1-107.6)	99.7 (88.5-116.6)	99.3 (83.7-106.7)	98.0 (88.1-125.0)	0.08^a^	0.44^a^
LVESVI (ml/m2)	51.3 (47.5-62.6)	55.1 (38.1-80.6)	63.1 (48.9-79.7)	64.1 (52.8-71.6)	61.1 (54.0-83.6)	*0.03*^a^	0.39^a^
LVMI (g/m2)	50.0 (47.4-55.7)	46.3 (42.8-67.3)	50.9 (43.2-56.7)	48.8(42.0-59.6)	58.0 (50.4-63.9)	0.24^a^	0.96^a^
LVEF (%)	42.0 ± 7.9	43.3 ± 7.5	36.5 ± 9.4	37.1 ± 10.0	35.0 ± 11.3	0.02	0.34
AAR (%LVM)	48.6 (35.9-66.5)	63.0 (49.7-65.3)	56.8 (37.6-67.7)	89.2 (77.2-98.1)	n/a	0.001	0.05
IS (%LVM)	25.4 ± 16.0	20.5 ± 12.5	39.8 ± 21.8	47.4 ± 22.7	23.8 ± 11.5	0.02^a^	0.33^a^
MVO presence (%)	26 (55.3%)	7 (58.3%)	5 (62.5%)	6 (100%)	15 (71.4%)	NS	--
MVO (%LVM)	0.5 (0.0-3.3)	0.2 (0.0-3.9)	1.2 (0.0-4.6)	6.4 (1.0-14.8)	1.3 (0.0-2.8)	0.08	0.37

PPCI = primary percutaneous coronary angioplasty, LVEDVI = left ventricular end-diastolic volume index, LVESVI = left ventricular end-systolic volume index, LVMI = left ventricular end-diastolic mass index, LVEF = left ventricular ejection-fraction, AAR = ischaemic area at risk (%LV mass), IS = infarct size (%LV mass), MSI = myocardial salvage index (%), MVO = microvascular obstruction (%LV mass).

^a^analysed using Log10 transformed data.

#### ***Volumes and function***

LV volumes were higher and LV ejection fraction lower in the late-PCI, R-PCI groups and non-reperfused cohorts compared with the PPCI and thrombolysed patients. In reperfused patients, when corrected for TTR, the differences in LVESVI and LVEF were no longer significant (Table 2).

#### ***IS, AAR and MVO***

IS differed across the five study cohorts, being higher in R-PCI and late PCI groups compared with PPCI and thrombolysed patients (late PCI vs PPCI p = 0.015, late PCI vs thrombolysis p = 0.008, late PCI vs non-reperfused p = 0.014, R-PCI vs thrombolysis p = 0.06 on subgroup analysis). When corrected for TTR, the differences in IS in reperfused patients were no longer statistically significant (p = 0.33).

**Figure 3:**
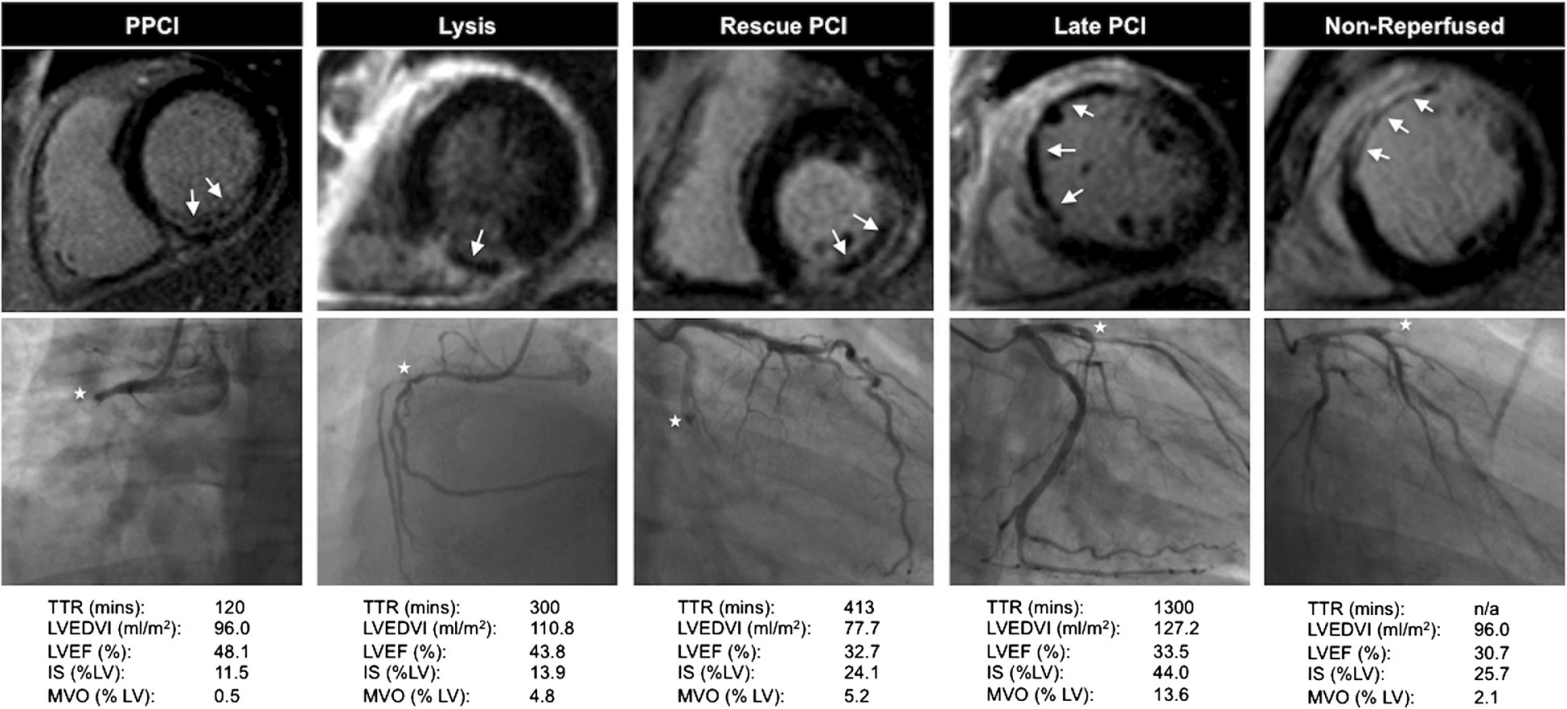
**Representative images of LGE CMR and coronary anatomy at the start of angiography in the cohorts. ***Top row*: CMR late gadolinium images from a patient within each of the 5 study cohorts, demonstrating infarct (enhancement); microvascular obstruction (arrow) evident as hypointense areas within infarct. *Middle row*: coronary angiography images at the start of angiography in the same patients demonstrating infarct related artery; white star denotes culprit lesion (right coronary artery in PPCI and lysis patient, left circumflex in rescue-PCI patient, left anterior descending artery in late PCI and non-reperfused patient). *Bottom row*: Time from symptoms to revascularisation (TTR) and CMR data for the same patients.

AAR was significantly larger in the late PCI group compared with those undergoing the 3 alternative reperfusion techniques (p < 0.01 compared with each strategy on subgroup analysis). When corrected for TTR, differences in AAR were only of borderline statistical significant (p = 0.054).The prevalence of MVO was similar in the 5 cohorts. There was a trend towards the extent (% of LV mass) of MVO being greatest in the late PCI group, followed by non-reperfused and R-PCI patients. When corrected for TTR, the difference in MVO with the four reperfusion techniques was not statistically significant. Representative CMR and angiography images from patients in the 5 cohorts are shown in Figure 3.

#### ***Interobserver and intraobserver agreement of CMR analysis of infarct characteristics***

Interobserver agreement for IS, MVO, AAR and MSI was excellent, with ICCs of 0.905, 0.958, 0.888 and 0.931 respectively. Intraobserver agreement was also excellent, with ICCs as follows: (a) observer 1: IS (0.980), MVO (0.984), AAR (0.946), MSI (0.937), and (b) observer 2: IS (0.991), MVO (0.991), AAR (0.948), MSI (0.982).

### Predictors of IS and MVO in patients receiving reperfusion therapy

In reperfused patients (n = 73), univariate predictors of IS were TTR, AAR, LAD IRA, reperfusion method, TIMI grade post-PCI and time from admission to CMR. In a stepwise multivariate model including all of the above plus TIMI grade pre-PCI, independent predictors of IS were TTR, AAR and TIMI grade post-PCI (model R^2^ = 0.41, Table 3).

**Table 3:** Predictors of Infarct Size (IS) in reperfused patients

**Dependent variable for IS**	**r**	**R**^**2**^	**B**	**p**
**Univariate**				
TTR (mins)^a^	0.47	0.21	26.17	<0.001
AAR (%LVM)	0.46	0.20	0.39	<0.001
LAD IRA	0.39	0.15	--	0.001
Reperfusion method	0.39	0.15	--	0.003
TIMI grade post-PCI	0.32	0.09	-8.23	0.006
Time from admission to CMR (d)	0.29	0.07	4.62	0.01
TIMI grade pre-PCI	0.15	0.01	-2.74	0.22
**Multivariate**				
*(Strongest model = TTR + AAR + LAD IRA + Reperfusion method + TIMI post + Time from admission to CMR):*		0.41		
TTR (mins)^a^			17.72	0.02
AAR (%LVM)			0.23	0.02
TIMI grade post-PCI			-5.21	0.04
LAD IRA			6.49	0.10
Time from admission to CMR (d)			2.54	0.12
Lysis v PPCI			-8.60	0.08
R-PCI v PPCI			6.45	0.27
Late PCI v PPCI			-7.05	0.45

PCI = percutaneous coronary intervention, TTR = time from symptom onset to revascularisation, AAR = ischaemic area at risk (%LV mass), IRA = infarct-related artery, LAD = left anterior descending artery, TIMI = thrombolysis in myocardial infarction, R-PCI = rescue PCI.

^a^analysed using Log10 transformed data.

Univariate predictors of MVO extent were TTR, AAR, reperfusion method and TIMI grade pre-PCI (Table 4). In a multivariate model including TTR, AAR, reperfusion method, TIMI-grade pre-PCI and TIMI-grade post-PCI, independent predictors of MVO were AAR, TIMI grade post-PCI and TTR (model R^2^ = 0.23, Table 4).

**Table 4:** Predictors of MVO extent in reperfused patients

**Dependent variable for MVO**	**r**	**R**^**2**^	**B**	**p**
**Univariate**				
TTR (mins)^a^	0.37	0.13	0.27	*0.001*
AAR (%LVM)	0.39	0.14	0.004	*0.001*
Reperfusion method	0.40	0.13	--	*0.008*
TIMI grade pre-PCI	0.35	0.08	-0.32	*0.03*
TIMI grade post-PCI	0.32	0.06	-0.98	0.06
LAD IRA	0.16	0.01	--	0.18
Time from admission to CMR (d)	0.12	0.01	0.02	0.33
**Multivariate**				
*(Strongest model = TTR + AAR + TIMI post)*		0.23		
AAR (%LVM)			0.003	*0.01*
TIMI grade post-PCI			-0.08	*0.03*
TTR (mins)^a^			0.16	*0.049*

PCI = percutaneous coronary intervention, TTR = time from symptom onset to revascularisation, IRA = infarct-related artery, LAD = left anterior descending artery, TIMI = thrombolysis in myocardial infarction, AAR = ischaemic area at risk (%LV mass).

^a^analysed using Log10 transformed data.

### IS and MVO in early v non-reperfused patients

The 59 patients receiving PPCI or successful lysis (<12 h) were grouped together as the ‘early-reperfused’ group for comparison with the non-reperfused group (n = 21) and results are shown in Table 5. LV volumes were higher and LVEF lower in the non-reperfused group compared to the early-reperfused group. IS was similar in the two groups despite CMR being performed later in the non-reperfused group. The prevalence and extent of MVO was similar in the two groups. Representative CMR and angiographic images from patients within our 5 study groups are shown in Figure 3.

**Table 5:** CMR data for early reperfused versus non-reperfused patients

**Variable**	**Early reperfused (n = 59)**	**Non-reperfused (n = 21)**	**p**
Age (y)	60.2 ± 11.9	65.6 ± 16.2	0.11
Male sex (%)	53 (89.8)	16 (76.2)	0.12
Time admission-CMR (d)	1.9 (1.2-2.6)	6.6 (4.8-11.0)	*<0.001*
LVEDVI (ml/m2)	90.7 (82.4-102.7)	98.0 (88.1-125.0)	*0.005*
LVESVI (ml/m2)	51.4 (45.4-62.6)	61.1 (54.0-83.6)	*0.002*
EF (%)	42.3 ± 7.8	35.0 ± 11.3	*0.002*
IS (%LVM)	24.4 ± 15.3	23.8 ± 11.5	0.87
MVO prevalence (n,%)	33 (55.9%)	15 (71.4%)	0.21
MVO (%LVM)	0.4 (0.0-2.9)	1.3 (0.0-2.8)	0.36

PPCI = primary percutaneous coronary angioplasty, LVEDVI = left ventricular end-diastolic volume-index, LVESVI = left ventricular end-systolic volume-index, LVEDMI = left ventricular end-diastolic mass, LVEF = left ventricular ejection fraction, IS = infarct size (%LV mass), MVO = microvascular obstruction (%LV mass).

## Discussion

Microvascular obstruction is widely regarded as a manifestation of reperfusion injury after STEMI [1-3,16,17]. Here, we demonstrate that MVO occurs frequently in all forms of reperfusion therapy for STEMI, but also in those presenting late, receiving no specific reperfusion therapy. Although IS and the extent of MVO appeared to be greatest in those receiving reperfusion late (R-PCI or late PCI]), this difference was not statistically significant when adjusted for TTR, an important determinant of IS [18,19] and prognosis [20] following PPCI. Indeed, there was a similar prevalence and trend towards increased extent of MVO in patients receiving no reperfusion therapy compared with those undergoing timely reperfusion. Our findings suggest that in real-life clinical patients presenting with STEMI, CMR-measured MVO is primarily an ischaemic injury rather than a reperfusion injury *per se*. This may have implications for currently planned and future trials in PPCI assessing therapies specifically designed to reduce reperfusion injury.

### CMR-MVO and reperfusion injury

‘No-reflow’ was first demonstrated in canine myocardium in 1974 [3], and is characterized by ultrastructural changes secondary to severe microvascular injury [1,16]. MVO is generally assumed to be primarily related to reperfusion injury [1-3,16,17]. Animal studies have demonstrated infarct expansion and an almost three-fold increase in MVO extent in the first 48 hours post reperfusion, and a corresponding reduction in regional blood-flow to <45% of that pre-ischaemia, after 2 minutes of hyperaemia [21,22]. Reperfusion has been postulated to contribute to MVO through embolization of debris [23], release of vasoconstrictor and inflammatory substances (e.g. serotonin, thromboxane-B) [24] and mechanical damage to the capillary bed [16].

MVO is visualised on CMR by first-pass perfusion, early gadolinium imaging and LGE imaging as hypoenhanced areas within infarct cores [9] and is seen in up to 60% of PPCI patients post STEMI [25]. LGE-derived MVO (‘late MVO’) is felt to be the most important measure of MVO because of its strong correlation with ST-segment resolution, adverse ventricular remodeling [5] and major adverse cardiovascular events [9,26]. In both experimental models [27] and in patients treated by PPCI there is a strong correlation between MVO extent and IS on CMR [28,29].

Consistent with an extensive evidence base demonstrating correlation between the duration of ischaemia (TTR) and the extent of myocardial injury, our non-reperfused cohort had larger LV volumes and lower LVEF [18,19] compared with those promptly reperfused. CMR was performed later in the non-reperfused group. The extent of IS and MVO measured by CMR is known to decrease during the first week following treatment for STEMI (IS: reduction of ~21-30% in humans [30,31]; MVO: reduction of ~48% in humans [30], ~67% in animals [32]). It is therefore likely that had CMR been undertaken at a similar time-point after admission in non-reperfused and early-reperfused patients, the extent of IS and MVO may have been significantly greater in the non-reperfused cohort. Importantly, the FWHM technique requires minor operator input and results in extremely high intra- and interobserver agreement for quantification of MVO.

Our data suggest that CMR-measured MVO should not be used as a surrogate of subclinical angiographic ‘no-reflow’ or as a specific marker of reperfusion injury. Reperfusion injury is one component contributing to overall IS, [16,17] but in real-world patients presenting typically 2–3 hours after symptom onset with STEMI, the contribution of reperfusion to overall injury may be impossible to assess. Our data clearly show that CMR-measured MVO is extremely prevalent in non-reperfused patients and like IS, is strongly related to TTR and AAR in those receiving reperfusion therapy. This finding casts doubt on the selection of MVO, as opposed to IS or myocardial salvage index, as the primary CMR-based outcome in clinical trials that specifically aim to reduce reperfusion injury. As TTR is strongly related to IS and MVO, the potential to ameliorate true reperfusion injury will be greatest in those who have less ischaemic injury at the time of P-PCI, and short duration of symptoms, e.g. <3 hours from symptom onset may be where the benefit of effective treatments will be realised [20,27,28].

### Myocardial and microvascular damage by revascularisation strategy

CMR characteristics were similar with PPCI and thrombolysis, consistent with Bodi who demonstrated no differences in LV volumes, LVEF, IS, MVO or myocardial salvage index (MSI) [11]. The small number of late-PCI and R-PCI patients make statistical comparisons difficult. Our observations are similar to Ruiz-Nodar who demonstrated only 9% MSI with R-PCI [33], and the MERLIN study demonstrated similar LV function at 30 days in R-PCI compared with conservatively treated patients [34]. The current evidence base demonstrates a lack of prognostic benefit with late PCI [35]. All late PCI patients in our study had LAD infarcts and tended to be younger, factors likely to influence the clinical decision to proceed to intervention. The LAD IRA is likely to account for their larger AAR. The effects of R-PCI and late PCI on reducing LV myocardial and microvascular damage in STEMI remain unclear.

### Limitations

Patients were not randomized. The non-reperfused group were retrospectively identified and underwent CMR later than patients receiving reperfusion, however this difference should underestimate both the prevalence and extent of MVO in this group. The numbers of patients being treated with late PCI and R-PCI are small and no definitive conclusions can be drawn on the infarct characteristics.

## Conclusions

CMR-derived MVO is highly prevalent in STEMI patients not receiving reperfusion therapy. CMR measured MVO is more closely related to ischaemic time than reperfusion therapy in STEMI and may not be a good surrogate marker of reperfusion injury.

## Competing interests

The authors declare that they have no competing interests.

## Authors’ contributions

GPM, IBS and AHG conceived the idea for the study and developed the protocol. NR and GPM recruited patients and were present at study visits. JNK and NR performed the CMR analyses. JNK performed the angiographic analysis. JNK and NGDM performed the statistical analysis. JNK wrote the paper, which all authors critically reviewed for content. All authors read and approved the final manuscript.
